# The Plague in India

**Published:** 1897-08

**Authors:** N. C. Mitra

**Affiliations:** Ranchi, Bengal, India


### CORRESPONDENCE.

---

### THE PLAGUE IN INDIA.

RANCHI, BENGAL, INDIA, June 10, 1897.

*Editor Atlanta Medical and Surgical Journal:*

I now send paper on the Plague, which I promised some time ago. Owing to pressing engagements I could not get it ready earlier.

On the 23d of September, 1896, Dr. Viegas, of Bombay, informed the municipal commissioners that he had treated some cases which were akin to those of plague. The matter was discussed at the municipal meeting. It was acknowledged by medical men that the disease had been in existence for a fortnight or so, and was characterized by the appearance of glandular swellings. The mortality was not of such an alarming nature as would lead men to associate the disease with plague. Dr. Surveyor, of Bombay, found Kitasato bacilli in the blood of the patients. The disease was diagnosed by some as a mild form of plague, by others as a form of fever, with glandular enlargements. Opinions differed. Some of the practitioners who had been in parts of Arabia where plague is an epidemic disease, gave it as their emphatic opinion that the disease was nothing else than plague. A conference of the local medical men was held and the disease prevailing at the time in Bombay was diagnosed as the plague appearing in the mildest form imaginable. In the meantime, so soon as the wire flashed the news to Calcutta, the city fathers convened meetings to protect the metropolis from the ravages of the disease. Stringent measures were adopted to cleanse and disinfect the dirtiest quarters, and passengers were thoroughly examined before their entrance into Calcutta. Owing to the adoption of these measures, not a single case of bubonic plague has occurred in the town.

Let us now turn our attention to Bombay, where the disease gradually wore a darker aspect, claiming its victims by hundreds and thousands. It was confined at first to the dingiest quarters, among a population who forgot the meaning of cleanliness. Bombay has a population of over 700,000 souls. The disease was in the commencement confined to the grain dealers, and naturally it was associated with filth and dirt. The affected quarters were then thoroughly cleansed and disinfected; but in spite of every attempt, the disease showed no tendency to decrease, but went on increasing. As time wore on the disease commenced spreading to other quarters of the town. From the town it extended its ravages to the neighboring districts. The government of India deputed Surgeon-General Cleghorn, M. Haffkine, of anti-cholera vaccination fame, and Mr. Hankin, an expert bacteriologist, to proceed to Bombay and find out the best means of combating and checking the spread of the disease. As all the methods hitherto adopted had not the slightest effect in preventing the spread, Dr. Cleghorn gave it out as his opinion that the only means was to have Bombay evacuated, the healthy separated from the diseased, whereas M. Haffkine wanted to have a military cordon drawn round Bombay and to have its connection cut off from the civilized world. As the latter proposal meant an immense loss to trade, it could not be carried out. Bombay having connection by sea with the principal ports of Europe, the whole of Europe got alarmed at the ravages of the plague, and some issued stringent orders enjoining quarantine on all vessels arriving from India. This finally lead to the Venice Sanitary Conference, in which all the European powers were represented. Dr. Cleghorn, too, sat there as the representative of the government of India. It was resolved, after much discussion, that ten days be fixed as the incubation period of the disease; and as it takes about a fortnight for vessels to arrive at the European ports from India, practically the passengers are quite free to land, unless there are cases of plague on board the vessel.

In spite of all efforts the disease showed no abatement. By this time (end of February) the ravages of the plague were at their maximum intensity, the daily deaths rising to 136. People were panic-stricken, and the town was being depopulated by tens of thousands. About 350,000 had already left Bombay, and germs of the disease got implanted in many of the neighboring districts. M. Haffkine commenced his prophylactic treatment, and Dr. Yersin was specially requested to come and try his anti-plague serum, which had worked wonders in Hongkong. In the meantime the government of India passed the Epidemic Diseases Act, in which segregation of the affected was made compulsory, and rigorous examination of passengers by rail from Bombay to other places was insisted on, and suspected cases detained in segregation camps. With the onset of the summer season the disease has showed decided tendency to decrease. General Gataere has been placed on duty at Bombay to carry out the provisions of the Epidemic Diseases Act and other sanitary measures that may be required. The daily mortality from the plague rose to a maximum in the Bombay city itself of 136; it has now declined to two. Considering the steady decline in the ravages of the disease, it is hoped the disease will die out in the summer months. It is quite impossible for experts even now to give a decided opinion as to whether there is any likelihood of any recrudescence of the disease in the rains. The most important point is the stamping out of the disease, as it is an inevitable result that, otherwise, it will take an epidemic form. The plague is an epidemic disease in certain parts of the Himalayas, as the Komaun, where the disease is known as "mahamari." About a century back plague prevailed to an alarming extent in some parts of the Bombay presidency. Experiments carried on by Messrs. Haffkine and Hankin have more or less proved that the germs of the disease die out in a week. The value of the prophylactic treatment of M. Haffkine, or of the curative one of Dr. Yersin, is still on trial, and no definite opinion can yet be expressed. We have at present centered in the Bombay district distinguished experts to study the disease with reference to it curative and prophylactic treatment. They are one and all concentrating their energies towards the solution of a problem which directly or indirectly affects the well-being of the whole civilized world.

*Causes.*—A specific bacillus is the cause of the disease. It was discovered by Kitasato, a Japanese medical man. It is found in the blood, fecal matter, and in the intestines.

The most important question is the origin of the plague in India. This point has till now puzzled the brain of even distinguished experts, that no definite opinion can be given. It may be well to enumerate these, leaving aside the question of *de novo* origin of the disease. It would be of distinct advantage if the disease could be traced to somewhere where the disease had been prevailing for some time. There was a severe epidemic of plague at Hongkong in 1894, and one in 1896, and plague is an endemic disease in parts of Arabia. The question was whether the plague bacilli could have found their way from any of these infected places to Bombay. Surgeon-Colonel Waters, of Bombay, does not believe in the theory of importation of the disease, and questions as to how the intervening parts, in that case, escaped from plague, and that Bombay, which was better in sanitation than the intervening stations, could be affected. And, moreover, the time occupied by vessels in coming from Hongkong would, to a certainty, kill these germs. Some were of the opinion that rats, the first to catch the infection, brought the disease to Bombay in bales of sugar and dates imported from Hongkong. How the bacilli got entry into Bombay is still a mystery. Plague bacilli have sometimes lived for a long time, as the following will show: In December, 1896, there were two cases of death from plague in the Seaman's Hospital at Greenwich, in England, and on inquiry it was found out that both the deceased had been in Bombay and had left it thirty-one days previously. These two cases show conclusively that the bacilli must have been alive for thirty-one days, and then able to start into fresh activity. These might have lain dormant in the system or in the clothes of the deceased. Moreover, there is a form of plague known as *pestis ambulans*, in which the bacilli resemble the true bacillus of Kitasato but which never develop the severe form. It is believed by some that these forms of plague are the precursors of the bubonic plague —the virulent form. And, indeed, Mr. Hankin informs that a

German investigator, working in Japan, has found the bacilli to remain alive on linen for more than a month. These considerations favor the view held by some that bacilli could have reached Bombay in a state of activity from Hongkong. Surgeon-Colonel Waters holds that in consideration of the fact that people living on rice or wheat were comparatively less attacked, and that those living on millet fared badly, he is of opinion that the origin of the plague in Bombay was the unearthing of old millet stored in damp cellars, and the fungi from these being let loose in the air, found in Bombay suitable nidus to grow and spread the disease. It must be mentioned that the disease first commenced among the grain dealers in the port trust estates of Bombay. Dr. Waters noticed that those living in the upper stories of the locality were the worst sufferers. This, he explains, was due to the fungi from old millet being disseminated through the air and consequently attacking those being in the upper houses. In further support of his theory, Dr. Waters mentions that plague, which is an epidemic disease in the Almorah District of the Himalayas, is ascribed by the people to millet grain stored in the ground.

*Contagion and Infection.*—That the disease is contagious, there is not the least doubt about it; that cases can be carried by touch, Dr. Lawson, of Hongkong, gives it as his opinion that skin-to-skin infection is impossible unless the one to be infected has some wound, but at Bombay cases have been seen in which the disease has been communicated to persons who had not any abrasions anywhere on the body, and that nothing except touch theory can be hazarded. Considering that people in the house in which the disease had broken out, and that people of the infected locality fall victims in numbers, the presumption in favor of the infective nature of the disease seems tenable. It is difficult to explain how some escape when they have been exposed to the grasp of the bacilli in all possible ways. Nor can we explain why the disease has not made extensive devastation in places where people of Bombay have removed. One thing is certain, that the bacilli must have suitable condition to favor their propagation, and unless this is favorable the bacilli lose their virulence and die out.

The incubation period is fixed at from three to six days.

*Symptoms.*—The fever ushers in with an ague. The skin becomes hot and dry. The tongue dry and cracked. There is early delirium. The eyes are bloodshot. The next day the bubo appears. There is then delirium and stupor. Coma follows, and the patient dies on the second or third day.

*Diagnosis.*—Dr. Viegas, of Bombay, relates how he saw cases with bubo succumbing in forty-eight hours, and how he at last diagnosed the cases as those of bubonic plague. He had to try hard to convince the Bombay municipal authorities that there were cases of plague. Some of the oldest practitioners diagnosed the cases as those of malignant fevers. Bacteriological diagnosis is the best test, but this, too, is sometimes misleading, as cases were seen in Calcutta in which the bacteriological examination of the blood by Dr. Simpson showed the typical bacilli. These cases were finally pronounced by expert bacteriologists not to be those of bubonic plague. This leads to the question whether there is any difference between the bacilli of mild cases *(pestis ambulans)* and of virulent types (bubonic plague), and are distinguishable under the microscope.

*Treatment* is more or less symptomatic. As the poison acts deleteriously on the heart, its action must be kept up and strengthened by requisite medicines, as digitalis and strychnine and alcohol. Anti-plague serum treatment is the most rational one, and time will show how far Drs. Haffkine and Yersin have succeeded in their labors. The only way to escape the ravages of the plague is to separate the healthy from the diseased. Lime washing is recommended by Dr. Waters. Recently there was a local outbreak of plague in Garwahl District, in the Himalayas, and the only means the people had to escape death was to evacuate the village and seek shelter in the jungles. In th[illegible] way the plague is put a stop to.

*Mortality.*—In the beginning 90 to 95 per cent. Laterly 85 to 90 per cent. In Hongkong it was 70 to 90 per cent. More than 10,000 persons died in Bombay city, and 28,000 in the Bombay presidency.

N. C. MITRA, A.M., M.D.

